# Protein Priming Followed by a Replication-Competent VSV-GP Vector Boost Induces Sustained Immune Control in Therapeutic Hepatitis B Vaccination

**DOI:** 10.3390/vaccines14030266

**Published:** 2026-03-16

**Authors:** Jinpeng Su, Anna D. Kosinska, Susanne Miko, Edanur Ates Öz, Dorothee von Laer, Janine Kimpel, Ulrike Protzer

**Affiliations:** 1 Institute of Virology, Technical University of Munich/Helmholtz Munich, 81675 Munich, Germany; jinpeng.su@tum.de (J.S.); anna.kosinska@tum.de (A.D.K.); susanne.miko@tum.de (S.M.); edanur.ates-oz@tum.de (E.A.Ö.); 2 German Center for Infection Research (DZIF), Munich Partner Site, 81675 Munich, Germany; 3 Institute of Virology, Medical University of Innsbruck, 6020 Innsbruck, Austria; virologie@i-med.ac.at (D.v.L.); janine.kimpel@i-med.ac.at (J.K.)

**Keywords:** vesicular stomatitis virus (VSV) vector, hepatitis B virus, chronic hepatitis B, therapeutic vaccination, viral vector, prime/boost vaccination

## Abstract

**Background/Objectives**: Eliciting robust immune responses against the hepatitis B virus (HBV) through therapeutic vaccination holds promise for curing chronic hepatitis B. We previously developed the heterologous protein prime/viral vector boost clinical vaccine candidate, *TherVacB*. Here, we evaluated a replication-competent chimeric vesicular stomatitis virus vector (VSV-GP) as an alternative viral vector boost vaccine. **Methods**: A recombinant VSV-GP vector co-expressing HBV surface and core antigens (VSV-GP-HBs/c) was generated and characterized for antigen expression. Its immunogenicity, antiviral efficacy, and durability were assessed in HBV-naïve and HBV-carrier mice, using protein primed, viral vector-primed, and multi-viral vector boost regimens. **Results**: VSV-GP-HBs/c efficiently expressed both HBV antigens in vitro. A single immunization with VSV-GP-HBs/c induced only weak HBV-specific immune responses in vivo. Replacing protein priming with VSV-GP-HBs/c resulted in modest immune activation and limited antiviral effects in HBV-carrier mice. In contrast, substituting the modified vaccinia virus Ankara (MVA)-HBs/c boost in the *TherVacB* regimen with VSV-GP-HBs/c elicited robust HBV-specific antibody responses and strong CD4 and CD8 T-cell immunity, assessed by intracellular IFN-γ staining after peptide stimulation. This regimen achieved a substantial reduction in serum HBsAg levels, numbers of HBV-positive hepatocytes, and intrahepatic HBV-DNA, with antiviral efficacy comparable to that of the classical *TherVacB* regimen. Notably, a second viral vector boost did not enhance HBV-specific immunity or antiviral efficacy; instead, it promoted dominant vector-specific CD8 T-cell responses. Long-term analyses performed 10 weeks after the last vaccination further demonstrated that a single protein-prime/VSV-GP-HBs/c boost was sufficient to achieve sustained antiviral control. **Conclusions**: These findings identify VSV-GP-HBs/c as an effective boost vector for therapeutic hepatitis B vaccination and establish protein priming followed by a single viral vector boost as an optimal strategy for sustained antiviral immunity.

## 1. Introduction

Chronic infection with the hepatitis B virus (HBV) remains a major global health challenge, affecting more than 250 million people worldwide and contributing substantially to morbidity and mortality, due to progressive liver disease, cirrhosis, and hepatocellular carcinoma [1]. Although current nucleos(t)ide analog therapies effectively suppress viral replication and mitigate disease progression, they rarely achieve a functional cure—defined as sustained loss of hepatitis B surface antigen (HBsAg) with or without seroconversion to anti-HBs [2,3,4]. As a result, most patients require lifelong treatment, underscoring the urgent need for therapeutic strategies that can restore long-term immune control of HBV.

A fundamental obstacle to curing chronic HBV infection is profound virus-specific immune tolerance. Persistent antigen exposure within the liver drives the functional exhaustion and deletion of HBV-specific CD8 T cells, impairs CD4 T-cell help, and promotes a tolerogenic intrahepatic environment that suppresses antiviral immunity [5]. Therapeutic vaccination aims to overcome these barriers by reactivating virus-specific immunity. To be successful, such vaccination must reconstitute coordinated HBV-specific humoral and cellular responses and, critically, induce functional cytotoxic T lymphocytes that are capable of eliminating HBV-infected hepatocytes [6,7].

Over the past two decades, multiple therapeutic HBV vaccine platforms, including protein subunit, DNA, and viral vector vaccines, have been evaluated [8,9]. However, most have shown limited efficacy in overcoming HBV-specific immune tolerance, T-cell exhaustion, and the strong immunoregulatory mechanisms in the liver [10,11]. Heterologous prime–boost vaccination has emerged as a promising approach to overcome these barriers. By sequentially applying distinct vaccine modalities, antigen presentation is optimized, and T-cell priming is improved. To this end, we have developed *TherVacB*, a therapeutic vaccine based on a heterologous prime–boost design that is currently under clinical evaluation. The regimen combines priming with adjuvanted recombinant virus-like particles (VLPs) HBsAg and HBV core antigen (HBcAg), and a boost using a modified vaccinia virus Ankara (MVA) vector encoding multiple HBV antigens. This coordinated antigen delivery has shown high efficacy in preclinical mouse models, where it elicited strong anti-HBs antibody responses, robust HBV-specific CD4 and CD8 T-cell immunity, and pronounced antiviral activity [12,13,14,15].

Vesicular stomatitis virus (VSV)-based vectors have gained attention as a highly immunogenic vaccine platform that is capable of inducing robust cellular immune responses [16,17]. Pseudotyping VSV with the glycoprotein of lymphocytic choriomeningitis virus (LCMV) generates a chimeric vector, VSV-GP, that retains strong immunostimulatory properties but lacks the neurotropism associated with wild-type VSV [18,19]. Notably, VSV-GP induces rapid and potent CD8 T-cell responses [20] and generates only limited vector-neutralizing immunity, allowing for repeated administration and flexible use in heterologous prime–boost regimens [18]. In contrast to replication-deficient vectors such as MVA, which do not amplify in mammalian cells, VSV-GP is replication-competent and enables transient in vivo antigen amplification, potentially enhancing effector T-cell priming [21]. The successful clinical use of recombinant VSV-based Ebola vaccines (rVSV-ZEBOV) demonstrates their translational capacity but also raises important considerations regarding viral shedding following vaccination [22,23,24]. Thus, beyond platform substitution, evaluating VSV-GP as a boost component addresses whether replication competence and reduced anti-vector immunity after a second application confer immunological advantages in therapeutic hepatitis B vaccination, where overcoming immune tolerance and restoring effective antiviral T-cell immunity are critical.

To apply a VSV vector in a therapeutic vaccine scheme, several key knowledge gaps remain. First, it is unknown whether VSV-GP can function effectively in the therapeutic setting of chronic hepatitis B, where constant antigen exposure, T-cell exhaustion, and hepatic immunoregulation may limit vector-mediated priming. Second, the optimal role of VSV-GP within heterologous vaccination regimens—whether as a priming vector, a boost component, or part of a multi-vector approach—has not been defined. Third, within the context of the *TherVacB* regimen, it remains unclear whether administering an additional viral vector vaccine would enhance the therapeutic benefit or, conversely, impair the antiviral efficacy by diverting immune responses toward vector antigens rather than HBV antigens. Addressing these questions is crucial for determining how VSV-GP can be optimally integrated into therapeutic hepatitis B vaccination strategies that shall overcome immune tolerance and reestablish antiviral immune control.

In this study, we generated a replication-competent VSV-GP vector co-expressing HBsAg and HBcAg (VSV-GP-HBs/c) and systematically evaluated its immunogenicity, antiviral activity, and functional role in heterologous prime–boost vaccination strategies. By assessing VSV-GP-HBs/c in protein-primed, viral vector-primed, and multi-vector boost regimens in both HBV-naïve and HBV-carrier mice, we define the conditions under which this vector achieves optimal immunological and antiviral effects. These findings provide mechanistic insights into how VSV-GP can be leveraged for therapeutic HBV vaccination, establishing a framework for its integration into next-generation curative strategies for chronic hepatitis B.

## 2. Materials and Methods

### 2.1. Generation of the VSV-GP-HBs/c Vector

The replication-competent VSV-GP vector co-expressing HBsAg and HBcAg (VSV-GP-HBs/c) was constructed by replacing the luciferase reporter gene at genome position 5 of the pVSV-GP-Luc backbone [25] with an HBsAg–HBcAg fusion sequence linked by a P2A peptide. The HBs/c fragment was excised from the original expression plasmid and inserted into the XhoI/NheI cloning site at position 5, under the control of the viral T7 promoter, generating the recombinant plasmid pVSV-GP-HBs/c. Recovery of the virus was achieved using an established VSV reverse-genetics system [26] by transfecting pVSV-GP-HBs/c, along with helper plasmids encoding the VSV N, P, M, L, and G proteins and T7 polymerase. The rescued virus was propagated in BHK-21 cells (ATCC, Manassas, VA, USA), plaque-purified, and expanded to produce working stocks. Supernatants were clarified, concentrated, aliquoted, and stored at −80 °C. Infectious titers were quantified by a TCID_50_ assay, as described previously [27].

### 2.2. Validation of Antigen Expression After VSV-GP-HBs/c Infection

Antigen expression following infection with VSV-GP-HBs/c was assessed by Western blot and immunofluorescence staining. For Western blot analysis, BHK-21 cells were infected with VSV-GP or VSV-GP-HBs/c for 40 h, before lysates were prepared under standard conditions. Proteins were denatured, clarified, and processed using the established Western blot procedures, described previously [14], with detection of HBsAg and HBcAg using specific primary antibodies. For immunofluorescence staining, BHK-21 cells grown on coverslips were infected with VSV-GP or VSV-GP-HBs/c and fixed at the designated time point. After permeabilization and blocking, cells were incubated with either HBsAg-specific monoclonal antibody (HB1) or HBcAg-specific polyclonal antibody (DAKO), followed by incubation with fluorophore-conjugated secondary antibodies. The fluorescence images were acquired using a Fluoview FV10i confocal microscope (Olympus, Tokyo, Japan) to visualize the antigen expression.

### 2.3. Ethical Statement

Animal experiments were conducted in strict accordance with the German regulations of the Society for Laboratory Animal Science (GV-SOLAS), and the European Guidelines for the Care and Use of Laboratory Animals of the Federation of European Laboratory Animal Science Associations (FELASA). The experimental protocols were reviewed and approved by the District Government of Upper Bavaria (approval numbers: 55.2-1-54-2532-103-12 and ROB-55.2-2532.Vet_02-18-24). Mice were housed in specific pathogen-free, biosafety level 2 facilities, in accordance with institutional guidelines. The study was designed and reported in compliance with the ARRIVE guidelines.

### 2.4. Animal Models

Wild-type C57BL/6J male mice (8–10 weeks of age) were obtained from Janvier Labs (Le Genest-Saint-Isle, France). To generate a model of persistent HBV replication, mice received an intravenous dose of 4–6 × 10^9^ genome equivalents (GE) of an adeno-associated virus (AAV)-HBV vector encoding a 1.2-fold overlength HBV genome (genotype D, ayw). AAV-HBV-infected, HBV-carrier mice were bled and stratified into experimental groups based on their circulating HBeAg (mean ~50 PEIU/mL) and HBsAg levels (30–1000 IU/mL, mean ~600 IU/mL), and then randomly assigned to different treatments. The sample sizes were determined based on prior experience with this HBV carrier mouse model (*n* ≥ 4). Investigators were not blinded during vaccine administration; however, outcome assessment and data analysis were performed in a blinded manner.

### 2.5. Vaccination Regimens in Mice

For single-dose immunization studies, wild-type C57BL/6 mice received 10^7^ TCID_50_ of VSV-GP or VSV-GP-HBs/c intramuscularly, and immune responses were analyzed one week later.

For prime–boost studies, vaccination schedules were adapted from the established *TherVacB* protocol [12]. In the standard *TherVacB* regimen, mice are primed twice with 10 µg each of particulate HBsAg (genotype A, adw) and HBcAg (genotype D, ayw) VLPs formulated with 10 µg c-di-AMP adjuvant, followed by a boost with 3 × 10^7^ infectious units (IFU) of recombinant MVA expressing HBV S and core antigens (MVA-HBs/c), with two-week intervals between immunizations. In the regimens evaluated in this study, 10^7^ TCID_50_ of VSV-GP-HBs/c was used either as an alternative priming vaccine to replace protein immunization or as a substitute or addition to the MVA vector boost. Vaccine-induced immune responses were assessed at defined time points, including one, six, or ten weeks after the final immunization. Details of individual vaccination schemes are provided in the corresponding figure legends.

### 2.6. Analysis of HBV Parameters in Murine Serum

Serum antigen levels were assessed using automated immunoassays on the Architect platform (Abbott Laboratories, Wiesbaden, Germany), as described previously [15]. Quantification of HBsAg and HBeAg was performed with the respective assay kits (HBsAg, Ref. 6C36-44; HBeAg, Ref. 6C32-27) and calibrated using the corresponding quantitative standards (Ref. 7P24-01). Anti-HBs antibodies were measured using the Architect anti-HBs assay (Ref. 7C18-27). Detection of anti-HBc antibodies was performed using the Enzygnost anti-HBc monoclonal assay (Siemens Healthcare Diagnostics, Erlangen, Germany).

### 2.7. Analysis of HBV Parameters in Murine Liver Tissue

Intrahepatic HBV-DNA levels were quantified from liver tissue extracts of HBV-carrier mice. DNA was isolated using a commercial extraction kit (NucleoSpin Tissue DNA Kit, Macherey-Nagel, Dueren, Germany), and HBV-DNA was measured by SYBR Green-based real-time PCR on a LightCycler 480 system (Roche, Mannheim, Germany). Amplification targeted a conserved region of the HBV genome (primers: HBV-1745-Fwd, 5′-GGAGGGATACATAGAGGTTCCTTGA-3′; HBV-1844-Rev, 5′-GTTGCCCGTTTGTCCTCTAATTC-3′), with results normalized to the single-copy prion protein (PrP) gene using established primer sets. The amplification conditions for both PCR reactions followed the PCR programs as described [28].

For the histological assessment of hepatic HBV antigen expression, the liver specimens were fixed in paraformaldehyde for 48 h and embedded in paraffin. Sections (2 μm) were stained for HBV core antigen, using previously validated immunohistochemical methods [29]. HBc-positive hepatocytes were quantified by counting stained cells in ten randomly selected fields at 20× magnification and normalizing the counts to tissue area (mm^2^).

### 2.8. Isolation of Lymphocytes and Detection of T-Cell Responses by Intracellular Cytokine Staining (ICS)

Murine splenocytes were isolated by mechanical dissociation through 100 µm strainers with subsequent erythrocyte lysis, using ammonium–chloride–potassium (ACK) buffer. Liver-associated lymphocytes (LALs) were prepared following established protocols [30].

For intracellular cytokine staining (ICS), cells were stimulated with overlapping peptide pools derived from HBsAg or HBcAg, or VSV- or MVA-specific peptides (2 µg/mL) in the presence of brefeldin A (1 µg/mL; Sigma-Aldrich, Taufkirchen, Germany) for 14 h; the ovalbumin (OVA)-derived SIINFEKL peptide served as a control stimulus. Following stimulation, surface markers were stained with anti-CD4 and anti-CD8 antibodies, and dead cells were excluded by using a fixable viability dye (eF780, eBioscience, Frankfurt, Germany). Cells were then fixed, permeabilized, and stained for intracellular IFN γ and TNFα. Samples were acquired on a CytoFLEX S flow cytometer (Beckman Coulter, Brea, CA, USA) and analyzed using FlowJo 10 software (BD Biosciences, Ashland, OR, USA).

### 2.9. Statistical Analysis

In all graphs, data are presented as mean ± SEM. Statistical evaluations were performed using GraphPad Prism 10 (GraphPad Software Inc., San Diego, CA, USA). Data were first assessed for normality using the Shapiro–Wilk test. Because most datasets did not follow a normal distribution, nonparametric statistical tests were used throughout. Comparisons between two groups were performed using the Mann–Whitney U test, while comparisons involving more than two groups were analyzed using the Kruskal–Wallis test, where applicable. A *p*-value < 0.05 was considered statistically significant. Only statistically significant differences are indicated in the figures.

## 3. Results

### 3.1. Generation and Characterization of the Novel VSV-GP-HBs/c Vaccine Vector

**Figure 1:**
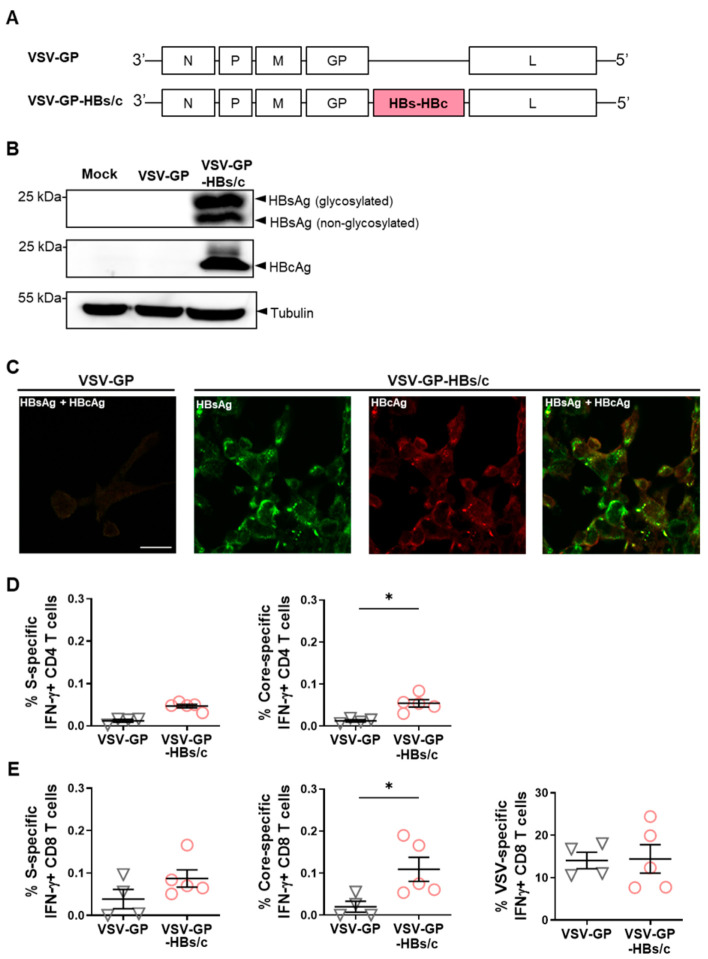
In vitro and in vivo characterization of recombinant VSV-GP-HBs/c. (**A**) Replication-competent vesicular stomatitis virus, pseudotyped with the glycoprotein of lymphocytic choriomeningitis virus (VSV-GP) expressing a HBs-HBc antigen cassette (HBs/c) at position 5 was constructed. (**B**) Expression of glycosylated and non-glycosylated HBsAg, HBcAg, and housekeeping protein Tubulin was detected by Western blotting BHK-21 cell lysates after infection with VSV-GP-HBs/c at an MOI of 0.1 for 40 h. BHK-21 cells infected with VSV-GP served as controls. (**C**) Immunofluorescence staining of HBsAg (green) and HBcAg (red) in VSV-GP and VSV-GP-HBs/c infected cells. Scale bar indicates 20 µm. (**D**,**E**) C57BL/6 mice were injected once with either 10^7^ TCID_50_ VSV-GP (*n* ≥ 4, black triangles) or VSV-GP-HBs/c (*n* ≥ 5, red circles) at week 0 intramuscularly. At week 1, the mice were sacrificed to analyze T-cell responses in splenocytes by intracellular cytokine staining (ICS) following stimulation with HBV S or core-specific peptide pools or VSV-specific NP52 peptide. (**D**) Percentages of HBV S- and core-specific IFNγ+ CD4 T cells. (**E**) Percentages of HBV S-, HBV core-, and VSV-specific IFNγ+ CD8 T cells. Mann–Whitney test, * *p* < 0.05. Only statistically significant differences are indicated. Original Western blots are presented in the Supplementary Materials.

To expand the flexibility and potency of the *TherVacB* heterologous prime–boost vaccine platform, we developed a replication-competent chimeric VSV-GP vector as an alternative to the original MVA vector component. Because both HBV S- and core-specific immune responses contribute to the antiviral activity of therapeutic HBV vaccines, an HBsAg–P2A–HBcAg (HBs/c) cassette was inserted into genomic position 5 of the pVSV-GP backbone between the GP and L genes (Figure 1A), to enable coordinated expression of both antigens. The resulting recombinant virus, VSV-GP-HBs/c, was rescued using an established reverse-genetics system and subsequently expanded and plaque-purified to generate working stocks.

Antigen expression from the vector was verified by Western blotting and immunofluorescence staining. HBsAg and HBcAg expression were detected in lysates from VSV-GP-HBs/c-infected BHK-21 cells, whereas neither antigen was detectable in mock- or empty-vector-infected cells; tubulin levels confirmed comparable protein loading across samples (Figure 1B). Consistent with the Western blot results, immunofluorescence staining confirmed robust HBsAg and HBcAg expression. In contrast, no antigen staining was observed in cells infected with the empty VSV-GP vector (Figure 1C).

To obtain an initial in vivo assessment of immunogenicity, HBV-naïve C57BL/6 mice were immunized once with VSV-GP-HBs/c or the empty vector. One week later, intracellular IFNγ staining of re-stimulated splenocytes revealed significantly higher frequencies of HBV core-specific CD4 and CD8 T cells in VSV-GP-HBs/c-immunized mice compared to those that received the empty VSV-GP vector vaccination. At the same time, S-specific responses were detectable but of a lower magnitude (Figure 1D,E). VSV-specific CD8 T-cell responses were comparably strong across groups, indicating that insertion of the HBV antigen cassette neither impaired vector fitness nor affected vector immunogenicity (Figure 1E).

Together, these data confirm the successful generation and amplification of the VSV-GP-HBs/c vector, demonstrate robust expression of both HBV antigens in vitro, and show that the vector elicits dominant vector immunity and only weak HBV-specific cellular immunity in HBV-naïve C57BL/6 mice after a single immunization. This indicated that incorporating VSV-GP-HBs/c into heterologous prime–boost therapeutic vaccination strategies may be promising.

### 3.2. Heterologous Prime–Boost Regimens Incorporating VSV-GP-HBs/c Elicit Robust Immune Responses in HBV-Naïve Mice

**Figure 2:**
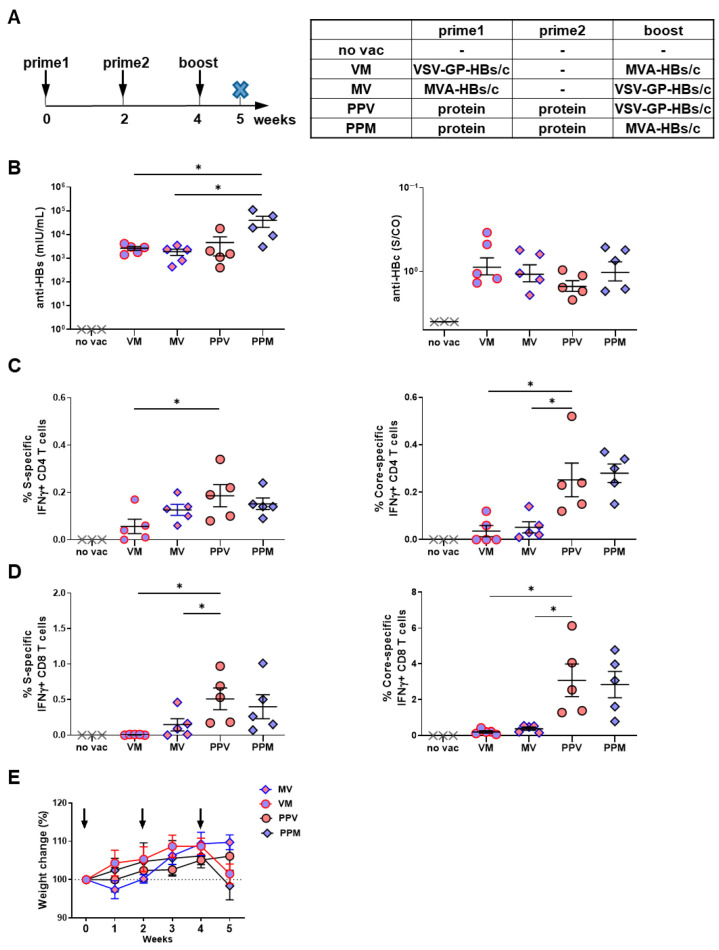
Immune responses induced by different VSV-GP-HBs/c-based heterologous prime–boost regimens in HBV-naïve mice. (**A**) Schematic depiction of vaccination regimens. Wild-type C57BL/6 mice were immunized according to the regimens shown in the table. Mice (*n* = 5) receiving a protein prime were vaccinated intramuscularly with HBsAg and HBcAg VLPs formulated with c-di-AMP at weeks 0 and 2. Mice (*n* = 5) receiving a viral vector prime were immunized intramuscularly with either 3 × 10^7^ IFU MVA-HBs/c or 10^7^ TCID_50_ VSV-GP-HBs/c at week 0. All groups were boosted at week 4 with either MVA-HBs/c (3 × 10^7^ IFU) or VSV-GP-HBs/c (10^7^ TCID_50_). One week after the boost (week 5), mice were sacrificed for analysis of vaccine-induced immune responses (indicated by the cross symbol × in the scheme). Mice without vaccination (*n* = 3) served as controls. (**B**) Serum levels of anti-HBs (**left** panel) and anti-HBc (**right** panel) at week 5. (**C**,**D**) Percentages of splenic S- (**left** panel) and core-specific (**right** panel) IFNγ+ CD4 (**C**) and CD8 T cells (**D**) were determined by ICS, following stimulation with HBV S- or core-specific peptide pools at week 5. (**E**) The weight of the mice was monitored weekly and compared with the baseline at the start of the experiment. Dash line indicates the baseline body weight of the mice. Arrows indicate the vaccination time points. Statistical analyses utilized the Kruskal–Wallis test, * *p* < 0.05. Only statistically significant differences are indicated.

To investigate the immunogenic potential of VSV-GP-HBs/c within heterologous prime–boost settings, we compared several vaccination regimens in HBV-naïve C57BL/6 mice. These included the classical *TherVacB* protein prime/MVA-boost strategy and modified regimens, in which VSV-GP-HBs/c was used either as an alternative boost or as a priming vector. Mice received two protein primes followed by a VSV-GP-HBs/c boost, or a viral vector prime (MVA-HBs/c or VSV-GP-HBs/c) followed by boosting with the other vector, respectively. Immune responses were analyzed one week after the final immunization (Figure 2A).

Immunization with all regimens induced substantial anti-HBs antibody levels exceeding 10^3^ mIU/mL. Protein priming resulted in higher anti-HBs titers than viral vector priming, with the classical protein/MVA *TherVacB* regimen eliciting significantly stronger anti-HBs responses than the vector-primed groups (Figure 2B, left panel). Antibody levels in the viral vector-priming regimens (MVA→VSV-GP and VSV-GP→MVA) were comparable, indicating that the vector administration sequence had no significant impact on the humoral immunity. Anti-HBc levels were uniformly high across all regimens, confirming the strong intrinsic immunogenicity of HBcAg [31] (Figure 2B, right panel).

However, the analysis of vaccine-induced T-cell responses revealed apparent differences between strategies. As expected, non-vaccinated mice did not exhibit detectable HBV-specific T-cell responses. Protein priming with c-di-AMP-adjuvanted VLPs induced the strongest splenic HBV S- and core-specific responses of IFNγ-producing CD4 T-cell, whereas viral vector priming resulted in markedly weaker helper T-cell immunity, particularly for core-specific responses (Figure 2C). These findings underscore the advantage of protein priming for eliciting robust Th1-type CD4 helper T-cell activation.

Interestingly, a similar pattern, with even more pronounced differences, was observed for CD8 T-cell responses. Protein priming with c-di-AMP adjuvanted VLPs followed by either VSV-GP-HBs/c or MVA-HBs/c induced the highest frequencies of splenic HBV S- and core-specific IFN-γ-producing CD8 T cells. In contrast, viral vector priming elicited significantly lower responses than the protein/VSV-GP-HBs/c regimen (Figure 2D). This highlights the superior capacity of protein priming to establish a strong cytotoxic T-cell foundation for subsequent vector boosting.

Finally, all vaccination regimens were well tolerated. While mice receiving MVA-containing regimens showed a transient and mild reduction in body weight, no sustained weight loss or clinical abnormalities were observed in any group, including those vaccinated with VSV-GP-HBs/c (Figure 2E).

Taken together, these results demonstrate that VSV-GP-HBs/c can be effectively integrated into heterologous prime–boost vaccination strategies and that protein priming followed by VSV-GP-HBs/c boosting elicits robust HBV-specific T-cell responses in HBV-naïve mice, which is comparable to those induced by an MVA-HBs/c boost.

### 3.3. Protein Priming Followed by VSV-GP-HBs/c Boosting Induces Strong Immune Responses in HBV-Carrier Mice

**Figure 3:**
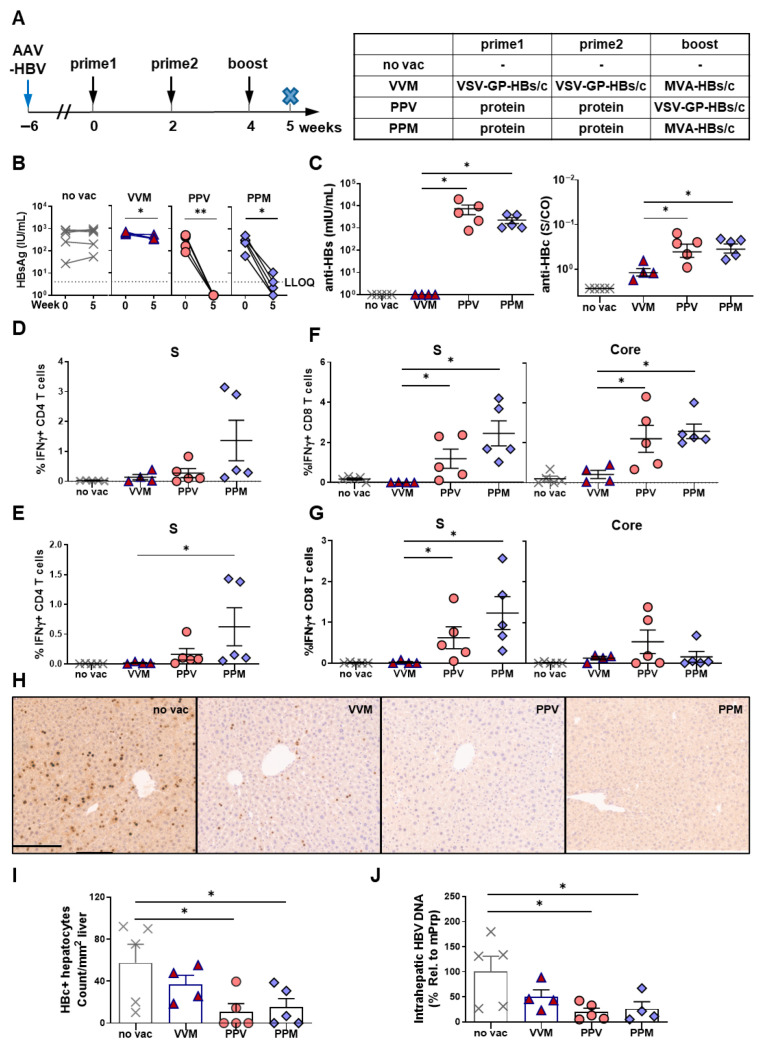
Immune responses induced by different VSV-GP-HBs/c-based heterologous prime–boost regimens in HBV-carrier mice. (**A**) Schematic depiction of vaccination regimens. C57BL/6 mice were intravenously injected with 6 × 10^9^ GE AAV-HBV1.2 six weeks before the start of vaccination to establish persistent HBV infection. The prime (weeks 0 and 2) and boost (week 4) immunization schemes are listed in the table. P = HBsAg and HBcAg proteins mixed and adjuvanted with c-di-AMP; V = 10^7^ TCID_50_ VSV-GP-HBs/c; M = 3 × 10^7^ IFU MVA-HBs/c. One week after boost immunization (week 5), mice were sacrificed to evaluate vaccine-induced immune responses (*n* = 5, except VVM *n* = 4). HBV-carrier mice that were not vaccinated served as controls (no vac). The cross-symbol x in the scheme indicates the terminal time point of the experiment. (**B**) Serum HBsAg levels at start (week 0) and endpoint (week 5). (**C**) Serum anti-HBs and HBc levels at week 5. (**D**,**E**) Percentages of S-specific IFNγ+ CD4 T cells in the liver (**D**) and spleen (**E**) were determined by ICS, following stimulation with S-specific peptide pools. (**F**,**G**) Percentages of S- and core-specific IFNγ+ CD8 T cells in the liver (**F**) and spleen (**G**) were determined by ICS, following stimulation with S- and core-specific peptide pools. (**H**) Representative images and (**I**) quantification of HBc-positive hepatocytes (brown) detected by liver immunohistochemistry staining. Scale bar indicates 100 µm. (**J**) Intrahepatic HBV-DNA detected in liver tissue lysates by qPCR. Statistical analyses used the Mann–Whitney test or Kruskal–Wallis test, * *p* < 0.05, ** *p* < 0.01. LLOQ, lower limit of quantification. Only statistically significant differences are indicated.

To evaluate the ability of VSV-GP-HBs/c to overcome HBV-specific immune tolerance, we next assessed the different heterologous prime–boost regimens in AAV-HBV-infected HBV-carrier mice, a model of persistent HBV replication. Based on the results obtained in HBV-naïve mice, the protein prime–VSV-GP-HBs/c boost regimen was directly compared with the classical *TherVacB* protein/MVA strategy. A viral vector priming regimen (two doses of VSV-GP-HBs/c followed by an MVA-HBs/c boost) was also included to explore whether protein priming could be replaced. Non-vaccinated (no vac) HBV-carrier mice served as controls (Figure 3A). All immunological and virological analyses were performed one week after the final boost immunization.

Irrespective of the viral vector used for boosting, protein-priming-based regimens led to a near-complete reduction in serum HBsAg levels by week 5, whereas VSV-GP-primed mice exhibited only a modest decline from baseline, comparable to the no-vac control in HBV-carrier mice (Figure 3B). Consistent with the decrease in serum antigen levels, mice primed with VSV-GP-HBs/c showed almost no detectable anti-HBs antibodies, while protein-primed mice developed high anti-HBs antibody levels, with no differences observed between those boosted with MVA-HBs/c or VSV-GP-HBs/c (Figure 3C). Anti-HBc antibody responses were robustly induced in all protein-primed groups, whereas the VSV-GP prime/MVA boost elicited significantly lower anti-HBc levels (Figure 3C).

To determine which of these regimens could break HBV-specific immune tolerance, HBV S- and core-specific T-cell responses were analyzed in liver-associated lymphocytes (LALs) and splenocytes. Protein priming regimens induced strong S-specific CD4 T-cell responses, with the PPM regimen yielding the highest frequencies in both the liver (Figure 3D) and the spleen (Figure 3E). Boosting with MVA-HBs/c elicited slightly stronger S-specific CD4 T-cell responses than boosting with VSV-GP-HBs/c. VSV-GP priming induced only low S-specific CD4 T-cell responses in either organ, despite the MVA boost (Figure 3D,E). Core-specific CD4 T-cell responses were barely detectable at the analyzed time point in both the liver and spleen of HBV-carrier mice (Supplementary Figure S1A,B). This indicated that the core-specific CD4 T-cell help was probably transient but functionally sufficient to promote antibody responses.

Protein priming also prepared the induction of robust CD8 T-cell immunity. Both intrahepatic and splenic S-specific CD8 T-cell responses were significantly elevated in PPM- and PPV-vaccinated mice, with a tendency toward higher responses after MVA-HBs/c boosting (left panels of Figure 3F,G). Likewise, protein priming generated strong core-specific CD8 T-cell responses in the liver after MVA and VSV-GP boosting (Figure 3F, right panel). In contrast, splenic core-specific CD8 T-cell responses were more pronounced in mice boosted with VSV-GP-HBs/c (Figure 3G, right panel). VSV-GP priming (VVM scheme) induced only low core-specific CD8 T-cell responses in the liver, which were not detectable in the spleen (right panels of Figure 3F,G).

To assess whether these immune responses translated into antiviral activity, liver sections were stained for HBV core protein, and intrahepatic HBV-DNA was quantified. Protein-primed groups, regardless of the vector used for boosting, displayed a substantial 60–70% reduction in the numbers of HBc-positive hepatocytes (Figure 3H,I) and a significant decrease in intrahepatic HBV-DNA levels (Figure 3J). In contrast, mice primed with VSV-GP-HBs/c exhibited only modest reductions compared to non-vaccinated controls (Figure 3H–J).

Taken together, these findings demonstrate that VSV-GP-HBs/c used for prime immunization was insufficient to break HBV-specific tolerance; however, protein priming followed by VSV-GP-HBs/c boosting induces strong HBV-specific humoral and cellular immunity, accompanied by robust antiviral effects in HBV-carrier mice. The magnitude of these responses was comparable to that achieved after boosting with MVA-HBs/c.

### 3.4. Protein Prime/VSV-GP-HBs/c Vector Boost Vaccination Achieves Long-Term HBV Control in HBV-Carrier Mice

**Figure 4:**
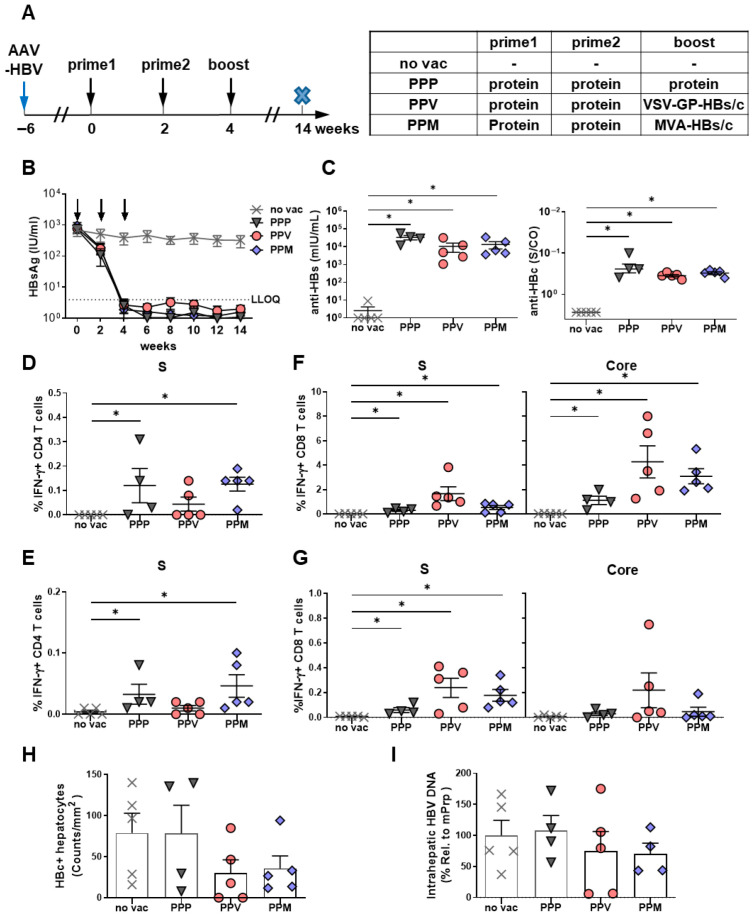
Long-term antiviral effects induced by different VSV-GP-HBs/c-based heterologous prime–boost regimens in HBV-carrier mice. (**A**) C57BL/6 mice were intravenously injected with 6 × 10^9^ GE AAV-HBV1.2 to establish persistent HBV infection before the start of prime/boost vaccination (week 6). The prime (weeks 0 and 2) and boost (week 4) immunization schemes are listed in the table. P = HBsAg and HBcAg proteins mixed and adjuvanted with c-di-AMP; V = 10^7^ TCID_50_ VSV-GP-HBs/c; M = 3 × 10^7^ IFU MVA-HBs/c. Then, 10 weeks after boost immunization (week 14), mice were sacrificed to evaluate vaccine-induced immune responses (*n* = 4, PPV: *n* = 5). HBV-carrier mice without vaccination served as controls (no vac, *n* = 5). The cross-symbol x in the scheme indicates the terminal time point of the experiment. (**B**) Time kinetics of serum HBsAg levels throughout the experiment. Arrows indicate vaccination time points. (**C**) Serum anti-HBs and HBc levels at week 14. (**D**,**E**) Percentages of S-specific IFNγ+ CD4 T cells in liver (**D**) and spleen (**E**) were determined by ICS, following stimulation with S-specific peptide pools. (**F**,**G**) Percentages of S- and core-specific IFNγ+ CD8 T cells in the liver (**F**) and spleen (**G**) were determined by ICS following stimulation with S- and core-specific peptide pools. (**H**) Quantification of HBc-positive hepatocytes (brown) detected by liver immunohistochemistry staining. (**I**) Intrahepatic HBV-DNA detected in liver tissue lysates by qPCR. Statistical analyses used the Mann–Whitney test or Kruskal–Wallis test, * *p* < 0.05. LLOQ, lower limit of quantification. Only statistically significant differences are indicated.

Building on the short-term analysis indicating that VSV-GP-HBs/c is a potent boost vector capable of breaking HBV-specific tolerance, we next investigated whether VSV-GP-HBs/c could also support the long-term immune control of HBV. HBV-carrier mice were primed twice with protein and subsequently boosted with either VSV-GP-HBs/c or MVA-HBs/c, and the immunogenicity and antiviral efficacy were assessed at week 14 (10 weeks after the boost immunization). In addition, a regimen consisting of three consecutive protein immunizations (PPP) was included to determine whether repeated protein administration alone can induce strong antibody responses and sustained antiviral T-cell immunity. Non-vaccinated mice served as the controls (Figure 4A).

PPM, PPV, and PPP groups receiving protein priming, including the three-protein regimen, showed a sustained, near-complete reduction in serum HBsAg. In contrast, HBsAg levels remained unchanged in the no-vac group (Figure 4B). Consistent with this antigen decline, all three vaccinated groups mounted significantly stronger anti-HBs and anti-HBc antibody responses than the no-vac controls (Figure 4C).

In contrast to the uniformly strong antibody responses, the durability and quality of HBV-specific T-cell immunity varied markedly across regimens. For S-specific CD4 T-cell responses, the PPP group developed CD4 T-cell frequencies that were comparable to those induced by the PPM regimen and higher than those induced by the PPV regimen in both the liver (Figure 4D) and the spleen (Figure 4E). HBV core-specific CD4 T cell responses were again barely detectable (Supplementary Figure S1C,D). These findings indicate that repeated protein administration is sufficient to establish robust and persistent S-specific CD4 T-cell responses.

However, this pattern was not reflected by CD8 T cell responses. The PPV regimen generated strong and long-lived intrahepatic and splenic S- and core-specific CD8 T-cell responses, comparable in magnitude to those induced by PPM (Figure 4F,G). In contrast, the PPP regimen elicited substantially lower HBV-specific CD8 T-cell responses in both the liver and the spleen (Figure 4F,G). Thus, although repeated protein immunization can effectively prime and maintain CD4 T-cell immunity, it fails to generate long-lasting cytotoxic CD8 T-cell responses. As expected, no HBV-specific T-cell responses were detected in non-vaccinated controls.

Both protein prime, vector boost regimens that induced strong CD8 T-cell responses, achieved sustained antiviral control, with more than a 60% reduction in HBc-positive hepatocytes (Figure 4H) and a marked decrease in intrahepatic HBV-DNA levels (Figure 4I), even ten weeks after the last immunization. In contrast, despite strong antibody responses and reduced serum HBsAg levels, the repeated protein immunization could not decrease the intrahepatic HBV-DNA levels or eliminate the HBc-positive hepatocytes, indicating that a reduction in serum HBsAg alone does not necessarily predict antiviral immune control (Figure 4H,I). These findings demonstrate that heterologous vaccination with protein priming followed by either MVA-HBs/c or VSV-GP-HBs/c boosting establishes sustained antiviral CD8 T-cell immunity and achieves long-term control of HBV in HBV-carrier mice.

### 3.5. Incorporation of a Second Viral Vector Boost Does Not Further Enhance the Efficacy of Therapeutic HBV Vaccination

**Figure 5:**
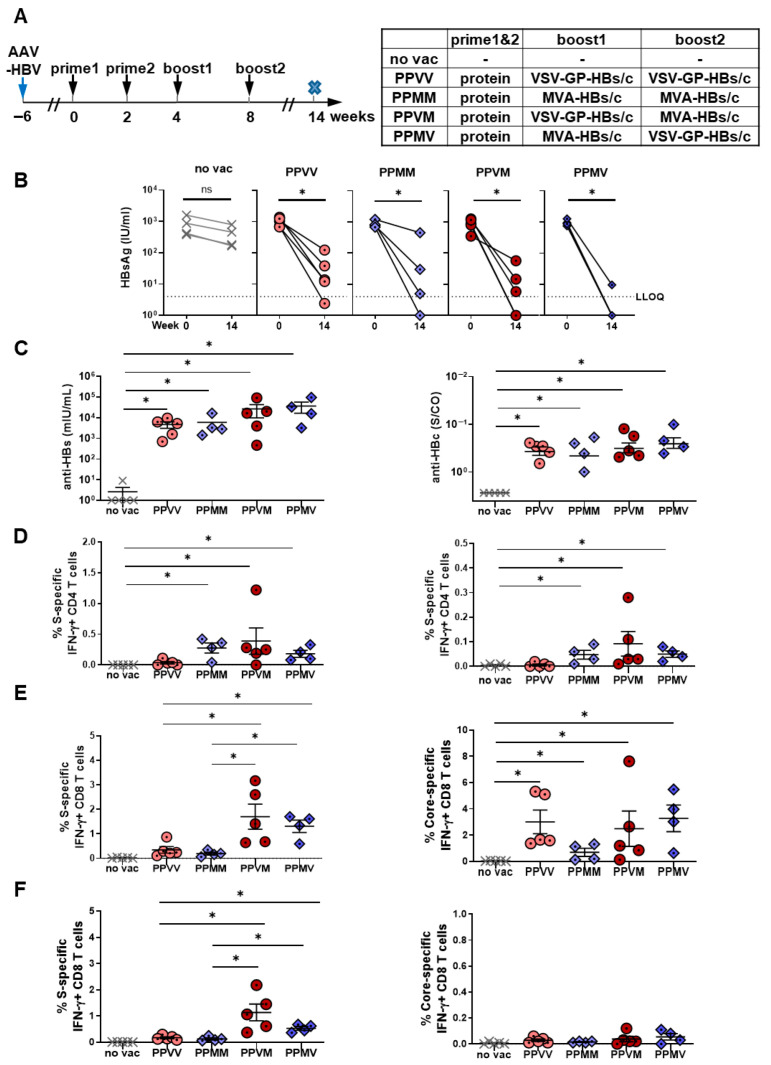
HBV-specific immune responses induced by twice protein priming—two viral vector boost regimens in HBV-carrier mice. (**A**) Schematic depiction of vaccination regimens. C57BL/6 mice were intravenously injected with 6 × 10^9^ GE AAV-HBV1.2 six weeks before the start of vaccination to establish persistent HBV infection. The prime (weeks 0 and 2) and boost (weeks 4 and 8) immunization schemes are listed in the table. P = HBsAg and HBcAg proteins were mixed and adjuvanted with c-di-AMP; V = 10^7^ TCID_50_ VSV-GP-HBs/c; M = 3 × 10^7^ IFU MVA-HBs/c (*n* = 4; PPVM: *n* = 5). Six weeks after the 2nd boost immunization (week 14), mice were sacrificed to evaluate vaccine-induced immune responses. HBV-carrier mice that were not vaccinated served as controls (no vac). The cross-symbol x in the scheme indicates the terminal time point of the experiment. (**B**) Serum HBsAg levels at start (week 0) and endpoint (week 14). (**C**) Serum anti-HBs and anti-HBc levels at week 14. (**D**) Percentages of S-specific IFNγ+ CD4 T cells in liver (left panel) and spleen (right panel) were determined by ICS, following stimulation with S-specific peptide pools. (E,F) Percentages of intrahepatic S- (left panel) and core-specific (right panel) IFNγ+ CD8 T cells were determined in liver (**E**) and spleen (**F**) by ICS, following stimulation with S- and core-specific peptide pools. Statistical analyses utilized the Mann–Whitney test or Kruskal–Wallis test, * *p* < 0.05. ns, not significant; LLOQ, lower limit of quantification. (**C**–**F**) Only statistically significant differences are indicated.

Given the strong boost of antiviral immunity induced by a single VSV-GP-HBs/c injection after protein priming, we wondered whether administering an additional viral vector boost could further enhance the therapeutic efficacy. HBV-carrier mice were primed twice with adjuvanted HBsAg/HBcAg VLPs and subsequently received two viral vector boosts—either twice with VSV-GP-HBs/c (PPVV), twice with MVA-HBs/c (PPMM), or one injection each of both vectors (PPVM, PPMV). As in the previous experiment, all immunological and virological analyses were performed at week 14 (Figure 5A).

HBsAg levels declined markedly in all two-vector boost combinations. However, in contrast to the near-complete HBsAg clearance observed in all mice after a single vector boost (Figure 4B), only a subset of mice in each two-vector boost group reached undetectable HBsAg levels (Figure 5B), despite the induction of high anti-HBs and anti-HBc antibody levels in these animals (Figure 5C). Analysis of longitudinal serum HBsAg levels revealed an initial decline after the first viral vector boost, followed by a partial rebound after the second boost (Supplementary Figure S2), explaining the limited HBsAg reduction observed at the final time point.

Analysis of HBV-specific CD4 T-cell immunity revealed comparable S-specific CD4 T-cell responses across the two-vector boost regimens in both the liver and the spleen, except for the PPVV group, in which responses were noticeably lower (Figure 5D). Quantitative analysis showed that a single viral vector boost elicited approximately 0.05–0.15% IFNγ+ HBV-specific CD4 T cells in the liver or spleen (Figure 4D,E), whereas two viral vector boosts increased these frequencies to approximately 0.1–0.3% (Figure 5D). HBV core-specific CD4 T cell responses were again barely detectable (Supplementary Figure S1E,F). These data indicate that prolonged antigenic stimulation provided by a second viral vector boost can further enhance HBV S-specific CD4 T-cell responses.

For CD8 T-cell immunity, PPVM and PPMV regimens induced significantly stronger S-specific CD8 T-cell responses than PPVV or PPMM, in both the liver (Figure 5E, left panel) and the spleen (Figure 5F, left panel). This indicated that sequential engagement of distinct viral vectors can more effectively stimulate cytotoxic T-cell activation. In contrast, all two-vector combinations following the protein prime generated comparable core-specific CD8 T-cell responses, suggesting that the order or type of viral vector administration has little influence on core-specific cytotoxic immunity. Notably, these core-specific CD8 T-cell responses were predominantly detected in the liver, the primary site of HBV infection (right panels of Figure 5E,F). When compared to the single-vector boost regimens shown in Figure 4, the magnitudes of both S- and core-specific CD8 T-cell responses remained similar, indicating that the addition of a second viral vector boost does not further strengthen HBV-specific CD8 T-cell immunity.

**Figure 6:**
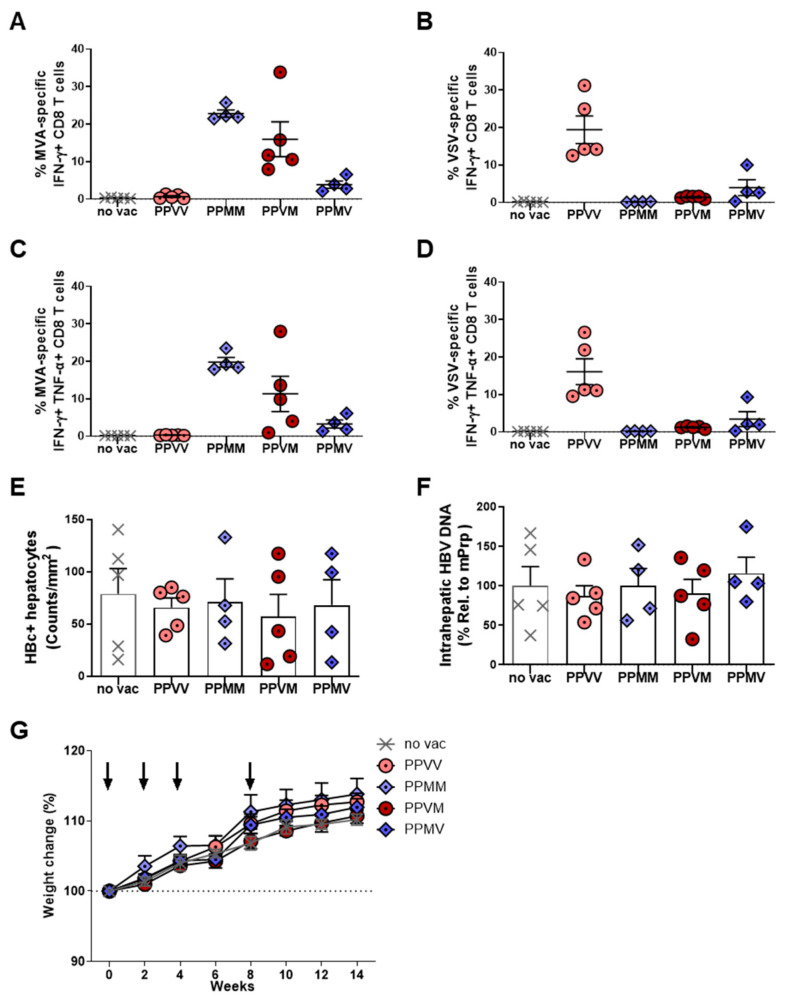
Virus-specific immune responses and antiviral effects induced by dual protein priming—dual viral vector boost regimens in HBV-carrier mice. C57BL/6 mice were infected with AAV-HBV and immunized as depicted in Figure 5A. End-point analyses were performed at week 14. (**A**–**D**) Percentages of vector-specific IFNγ+ and IFNγ+ TNFα+ CD8 T cells in the liver were determined by ICS following stimulation with MVA-specific B8R peptide (**A**,**C**) or VSV-specific NP52 peptide (**B**,**D**). (**E**) Quantification of HBc-positive hepatocytes (brown) detected by liver immunohistochemistry staining. (**F**) Intrahepatic HBV-DNA detected in liver tissue lysates by qPCR. (**G**) The weight of the mice in each group was monitored every 2 weeks and compared with the baseline values at the start of the experiment. Dash line shows the baseline body weight of the mice. Arrows indicate the vaccination time points.

Robust vector-specific CD8 T-cell responses against MVA but also against VSV-GP were detected, particularly following the second viral vector injection (Figure 6A,B). A multifunctional IFNγ+ TNFα+ CD8 T-cell response against immune-dominant vector peptides was induced and directed against both vectors, MVA and VSV, when the vector was administered twice (Figure 6C,D). Vector-specific CD8 T cell immunity was less prominent when the vector was administered only once and more dominant against MVA than against VSV-GP. Notably, despite the polyfunctionality, the pronounced anti-vector responses were not associated with enhanced HBV-specific immunity or a detectable antiviral efficacy. This indicates that repeated viral vector administration preferentially reinforces vector-specific, rather than HBV-specific, cytotoxic immunity.

Consistent with these immunological findings, assessment of antiviral efficacy revealed that neither of the two viral vector boost regimens conferred additional control of HBV replication. The number of HBc-positive hepatocytes remained comparably high in all dual-boost vaccination schemes applied (Figure 6E), and no reductions in intrahepatic HBV-DNA levels were detected (Figure 6F). Notably, the outcome was inferior to the antiviral effects achieved by a single viral vector boost in the preceding experiment (Figure 4), supporting the notion that enhanced vector-specific immunity may functionally limit the antiviral activity of HBV-specific CD8 T cells after repeated viral vector boosting. Concerning vector tolerability, body weight in all vaccinated groups remained stable or even increased over the long-term study period, confirming that repeated viral vector administration was well tolerated (Figure 6G).

Overall, these data demonstrate that incorporating a second viral vector boost provides no therapeutic advantage and can even compromise antiviral efficacy by inducing predominantly anti-vector immunity. This underscores that a single MVA-HBs/c or VSV-GP-HBs/c boost is both sufficient and preferable for achieving maximal immune and antiviral responses in therapeutic HBV vaccination.

## 4. Discussion

In this study, we developed and evaluated a replication-competent VSV-GP-HBs/c vector and examined its suitability as a boost component in therapeutic hepatitis B vaccination. When combined with protein priming, VSV-GP-HBs/c induced strong HBV-specific cellular immunity, broke HBV-specific tolerance in carrier mice, and achieved long-term antiviral control. Importantly, our findings further define the optimal use of this vector by showing that a single VSV-GP-HBs/c boost is sufficient and preferable. In contrast, additional viral vector boosting does not enhance—and may even impair—therapeutic efficacy.

The first key finding is that VSV-GP-HBs/c can replace MVA-HBs/c as the boost vector within the *TherVacB* regimen. After protein priming, both viral vectors triggered vigorous HBV-specific CD8 T-cell responses, particularly in the liver, and reduced serum HBsAg, intrahepatic HBV-DNA, and the numbers of HBV-positive hepatocytes. The extent and durability of these effects were comparable between VSV-GP-HBs/c and MVA-HBs/c, aligning with previous reports on the immunogenicity of MVA-based therapeutic HBV vaccines [8,15,32,33].

Although recombinant viral vectors are known to elicit strong CD8 T-cell responses [34], our results highlight the essential limitations of viral vector-only vaccination in the context of chronic HBV infection. A single VSV-GP-HBs/c immunization produced only weak HBV-specific responses, and, unexpectedly, even heterologous vaccination with two different viral vectors (VSV-GP-HBs/c and MVA-HBs/c) did not yield substantial HBV-specific CD8 T-cell immunity. This contrasts with earlier work, which shows that VSV-based vectors are highly effective in prophylactic settings, such as RSV and Ebola vaccination, where a single immunization can induce robust protective immunity [16,18,23,35]. The discrepancy highlights the fundamental distinction between prophylactic vaccination against other viral infections and therapeutic vaccination in chronic HBV infection, where the immune tolerance, T-cell exhaustion, and hepatic regulatory mechanisms impede effective priming [7,36].

Our findings, therefore, reinforce the central role of protein priming in therapeutic HBV vaccination. In both short- and long-term experiments, protein priming was essential for generating strong antibody responses and CD4 T-cell help, which in turn supported the expansion and maintenance of HBV-specific CD8 T cells after vector boosting. This finding is consistent with prior studies demonstrating that CD4 T-cell help is necessary for viral clearance during HBV infection [37,38]. Mechanistically, VLP-based priming provides advantages that are particularly critical in the setting of chronic HBV infection. Adjuvanted VLP-based vaccines promote longer-term antigen availability and efficient uptake by professional antigen-presenting cells. This allows for processing as an exogenous antigen and provides optimal conditions for optimal CD4 T-cell priming and helper cell differentiation [39,40]. Robust CD4 T-cell help is required for dendritic-cell licensing and for the effective expansion, functional maturation, and long-term maintenance of antiviral CD8 T-cell responses [41,42].

In contrast, viral vectors express antigens in a highly inflammatory but transient manner and promote endogenous antigen processing. They also induce a dominant vector-directed immune response, which can limit HBV-specific immunity through immunodominance and competition for antigen presentation [43,44,45]. These limitations are likely amplified in chronic HBV infection, where the tolerogenic hepatic environment and continuous antigen exposure further constrain effective priming of HBV-specific T-cell [46]. Notably, this interpretation is supported by our recent findings showing that proper protein priming programs the functional quality and hepatic recruitment of HBV-specific T cells and determines the overall efficacy of therapeutic HBV vaccination [14].

Another important observation from our findings is the dissociation between antibody responses and antiviral control. Multiple protein immunizations resulted in marked reductions in serum HBsAg and elicited strong anti-HBs responses; however, these regimens failed to induce long-lasting CD8 T-cell immunity or reduce intrahepatic viral parameters. In contrast, protein priming followed by MVA-HBs/c or VSV-GP-HBs/c boosting supported long-term cytotoxic T-cell responses and sustained antiviral effects. These results are consistent with clinical and experimental evidence demonstrating that antibody responses alone do not eliminate HBV-infected hepatocytes, and that CD8 T-cell-mediated immunity is central to achieving a functional cure and viral clearance [47,48,49].

The durability of immune responses achieved with VSV-GP-HBs/c is particularly relevant for therapeutic vaccination. Even ten weeks after the final immunization, protein/VSV-GP-HBs/c vaccination maintained strong intrahepatic CD8 T-cell responses and suppressed HBV replication. This is notable, given the tendency of chronic HBV infection to drive T-cell dysfunction through continuous antigen exposure and hepatic regulatory mechanisms [50].

A further key aspect of this study was the evaluation of repeated viral vector boosting. Although a second viral vector boost increased HBV-specific CD4 T-cell responses, it did not strengthen HBV-specific CD8 T-cell immunity or improve antiviral outcomes. Instead, repeated application of both vectors produced strong vector-specific CD8 T-cell responses. Heterologous boosting with first VSV-GP and then MVA yielded comparable CD8 T cell responses against both vectors, whereas boosting with MVA first and then VSV-GP elicited a dominant anti-MVA response. Polyfunctionality analyses further demonstrated that vector-specific CD8 T cells remained functionally active, indicating that the lack of improved antiviral efficacy was not due to global T-cell dysfunction. These findings support the view that therapeutic HBV vaccination requires careful coordination of priming and boosting, rather than simply increasing antigen or vector doses [5,6].

The preferential expansion of vector-directed immunity likely limits the functional capacity of HBV-specific cytotoxic T cells, a phenomenon described for adenoviral and poxviral vectors and attributed to immunodominance and vector-specific memory inflation [51,52,53]. Functionally, vector immunodominance can restrict therapeutic efficacy by competing for antigen presentation, costimulatory signals, inflammatory cytokines, and tissue niches required for HBV-specific CD8 T-cell expansion and effector differentiation [54,55,56]. However, we did not directly assess antigen presentation dynamics, exhaustion marker expression, or resource competition in the present study. Alternative mechanisms, such as differences in innate activation or antigen persistence following repeated vector exposure, may also contribute to the observed response hierarchy. Such competition is particularly detrimental in chronic HBV infection, where antiviral CD8 T cells are already numerically reduced and functionally exhausted, and where excessive vector-driven activation may further skew immune resources away from virus-specific responses [57,58].

A limitation of this study is the use of the AAV-HBV mouse model, which does not fully recapitulate all aspects of chronic HBV infection in humans. In this model, HBV replication is driven by hepatocyte transduction with AAV-HBV, rather than natural viral spread, and reinfection of hepatocytes does not occur [59]. Consequently, a viral rebound or reinfection under partial immune control cannot be fully assessed in this system and may be underestimated. Moreover, the circulating HBsAg levels achieved in our experiments may not reflect the levels in all patients with chronic hepatitis B, particularly during immune-tolerant phases of infection. While this model allows for controlled analysis of immune reactivation, the antiviral effects observed here should be interpreted as proof-of-concept, rather than a direct surrogate for clinical efficacy in patients with high antigen burden.

Safety is a key consideration for therapeutic vaccination using replication-competent viral vectors. In this study, VSV-GP-HBs/c was well tolerated, with no evidence of increased morbidity, mortality, or sustained weight loss across vaccination regimens. Mild and transient weight changes were observed in some groups, which was comparable to those reported for other viral vectors, including MVA, and resolved without clinical sequelae. No signs of neurological disease or systemic toxicity were detected, consistent with previous reports showing that pseudotyping VSV with the LCMV glycoprotein markedly reduces neurotropism and pathogenicity compared with wild-type VSV [18,19]. Systematic histopathological examination of non-hepatic tissues was not performed in the present study. Although detailed histopathological analyses were beyond the scope of this study, prior work has demonstrated a favorable safety and biodistribution profile for VSV-GP vectors, including upon repeated administration [18]. Together with the established clinical safety of the replication-competent rVSV-ZEBOV Ebola vaccine [22,23], these findings support the cautious further evaluation of VSV-GP as a boost component for therapeutic HBV vaccination. Nevertheless, comprehensive preclinical and safety studies will be required before clinical translation. However, one must consider that the transgene capacity of VSV-GP is limited and cannot express multiple antigens, unlike MVA vectors, which allow for the insertion of multi-antigen cassettes [15].

## 5. Conclusions

In summary, this study identifies VSV-GP-HBs/c as an interesting candidate boost vector for therapeutic hepatitis B vaccination. When combined with protein priming, it can break HBV-specific immune tolerance, elicit long-lasting cytotoxic T-cell responses, and achieve sustained antiviral control. At the same time, our data indicate that a single vector boost is sufficient and that repeated vector dosing does not offer any additional advantage. These results provide a strong rationale for the continued development of VSV-GP-based therapeutic vaccines and offer key insights into the design principles required to achieve a functional cure of chronic hepatitis B.

## Data Availability

The data presented in this study are available from the corresponding author upon reasonable request.

## Supplementary Materials

The following supporting information can be downloaded at: https://www.mdpi.com/article/10.3390/vaccines14030266/s1, Supplementary Figure S1: HBV core-specific CD4 T-cell responses induced by the immunization with different VSV-GP-HBs/c-based heterologous prime-boost regimens in HBV-carrier mice. Supplementary Figure S2: Time kinetics of serum HBsAg levels throughout the experiment.

## Abbreviations

The following abbreviations are used in this manuscript:

AAV	adeno-associated virus
ACK buffer	ammonium–chloride–potassium buffer
anti-HBc	hepatitis B core antibodies
anti-HBs	hepatitis B surface antibodies
ANOVA	Analysis of Variance
c-di-AMP	Cyclic di-adenosine monophosphate
GE	genome equivalents
HBV	hepatitis B virus
HBcAg	hepatitis B core antigen
HBeAg	hepatitis B e antigen
HBsAg	hepatitis B surface antigen
ICS	intracellular cytokine staining
IFN	interferon
IFU	infectious units
LALs	liver-associated lymphocytes
LCMV	lymphocytic choriomeningitis virus
LLOQ	lower limit of quantification
MVA	modified vaccinia Ankara virus
ns	not significant
no vac	non-vaccinated
OVA	ovalbumin
TCID_50_	tissue culture infection dose 50
TherVacB	therapeutic hepatitis B vaccine
TNF	tumor necrosis factor
WHO	World Health Organization
VLPs	virus-like particles
VSV	vesicular stomatitis virus
VSV-GP	a vector generated by pseudotyping VSV with the glycoprotein of LCMV

## References

[1] Hepatitis B Fact Sheet. https://www.who.int/news-room/fact-sheets/detail/hepatitis-b.

[2] Revill P., Testoni B., Locarnini S., Zoulim F. (2016). Global strategies are required to cure and eliminate HBV infection. Nat. Rev. Gastroenterol. Hepatol..

[3] Lok A.S., Zoulim F., Dusheiko G., Ghany M.G. (2017). Hepatitis B cure: From discovery to regulatory approval. Hepatology.

[4] Wong G.L., Gane E., Lok A.S. (2022). How to achieve functional cure of HBV: Stopping NUCs, adding interferon or new drug development?. J. Hepatol..

[5] Bertoletti A., Ferrari C. (2016). Adaptive immunity in HBV infection. J. Hepatol..

[6] Maini M.K., Burton A.R. (2019). Restoring, releasing or replacing adaptive immunity in chronic hepatitis B. Nat. Rev. Gastroenterol. Hepatol..

[7] Gehring A.J., Protzer U. (2019). Targeting Innate and Adaptive Immune Responses to Cure Chronic HBV Infection. Gastroenterology.

[8] Boni C., Barili V., Acerbi G., Rossi M., Vecchi A., Laccabue D., Penna A., Missale G., Ferrari C., Fisicaro P. (2019). HBV immune-therapy: From molecular mechanisms to clinical applications. Int. J. Mol. Sci..

[9] Kosinska A.D., Bauer T., Protzer U. (2017). Therapeutic vaccination for chronic hepatitis B. Curr. Opin. Virol..

[10] Ye B., Liu X., Li X., Kong H., Tian L., Chen Y. (2015). T-cell exhaustion in chronic hepatitis B infection: Current knowledge and clinical significance. Cell Death Dis..

[11] Knolle P.A., Huang L.-R., Kosinska A., Wohlleber D., Protzer U. (2021). Improving therapeutic vaccination against hepatitis B—Insights from preclinical models of immune therapy against persistent hepatitis B virus infection. Vaccines.

[12] Backes S., Jager C., Dembek C.J., Kosinska A.D., Bauer T., Stephan A.S., Dislers A., Mutwiri G., Busch D.H., Babiuk L.A. (2016). Protein-prime/modified vaccinia virus Ankara vector-boost vaccination overcomes tolerance in high-antigenemic HBV-transgenic mice. Vaccine.

[13] Michler T., Kosinska A.D., Festag J., Bunse T., Su J., Ringelhan M., Imhof H., Grimm D., Steiger K., Mogler C. (2020). Knockdown of Virus Antigen Expression Increases Therapeutic Vaccine Efficacy in High-Titer Hepatitis B Virus Carrier Mice. Gastroenterology.

[14] Su J., Brunner L., Oz E.A., Sacherl J., Frank G., Kerth H.A., Thiele F., Wiegand M., Mogler C., Aguilar J.C. (2023). Activation of CD4 T cells during prime immunization determines the success of a therapeutic hepatitis B vaccine in HBV-carrier mouse models. J. Hepatol..

[15] Kosinska A.D., Kächele M., Kerth H.A., Mück-Häusl M., Öz E.A., Gültan M., Hansen-Palmus L., Sacherl J., Ko C., Festag J. (2025). MVA-HBVac—A novel vaccine vector that allows pan-genotypic targeting of hepatitis B virus by therapeutic vaccination. Mol. Ther. Nucleic Acids.

[16] Rose N.F., Marx P.A., Luckay A., Nixon D.F., Moretto W.J., Donahoe S.M., Montefiori D., Roberts A., Buonocore L., Rose J.K. (2001). An effective AIDS vaccine based on live attenuated vesicular stomatitis virus recombinants. Cell.

[17] Wu F., Fan X., Yue Y., Xiong S., Dong C. (2014). A vesicular stomatitis virus-based mucosal vaccine promotes dendritic cell maturation and elicits preferable immune response against coxsackievirus B3 induced viral myocarditis. Vaccine.

[18] Tober R., Banki Z., Egerer L., Muik A., Behmüller S., Kreppel F., Greczmiel U., Oxenius A., von Laer D., Kimpel J. (2014). VSV-GP: A potent viral vaccine vector that boosts the immune response upon repeated applications. J. Virol..

[19] Muik A., Kneiske I., Werbizki M., Wilflingseder D., Giroglou T., Ebert O., Kraft A., Dietrich U., Zimmer G., Momma S. (2011). Pseudotyping vesicular stomatitis virus with lymphocytic choriomeningitis virus glycoproteins enhances infectivity for glioma cells and minimizes neurotropism. J. Virol..

[20] Travieso T., Li J., Mahesh S., Mello J.D.F.R.E., Blasi M. (2022). The use of viral vectors in vaccine development. npj Vaccines.

[21] Alharbi N.K. (2019). Poxviral promoters for improving the immunogenicity of MVA delivered vaccines. Hum. Vaccines Immunother..

[22] Agnandji S.T., Huttner A., Zinser M.E., Njuguna P., Dahlke C., Fernandes J.F., Yerly S., Dayer J.-A., Kraehling V., Kasonta R. (2016). Phase 1 trials of rVSV Ebola vaccine in Africa and Europe. N. Engl. J. Med..

[23] Henao-Restrepo A.M., Camacho A., Longini I.M., Watson C.H., Edmunds W.J., Egger M., Carroll M.W., Dean N.E., Diatta I., Doumbia M. (2017). Efficacy and effectiveness of an rVSV-vectored vaccine in preventing Ebola virus disease: Final results from the Guinea ring vaccination, open-label, cluster-randomised trial (Ebola Ça Suffit!). Lancet.

[24] Lee A.W., Liu K., Lhomme E., Blie J., McCullough J., Onorato M.T., Connor L., Simon J.K., Dubey S., VanRheenen S. (2024). Immunogenicity and vaccine shedding after 1 or 2 doses of rVSVΔG-ZEBOV-GP Ebola vaccine (ERVEBO^®^): Results from a phase 2, randomized, placebo-controlled trial in children and adults. Clin. Infect. Dis..

[25] Dold C., Urbiola C.R., Wollmann G., Egerer L., Muik A., Bellmann L., Fiegl H., Marth C., Kimpel J., Von Laer D. (2016). Application of interferon modulators to overcome partial resistance of human ovarian cancers to VSV-GP oncolytic viral therapy. Mol. Ther.-Oncolytics.

[26] Marzi A., Feldmann H., Geisbert T.W., Falzarano D. (2011). Vesicular stomatitis virus-based vaccines for prophylaxis and treatment of filovirus infections. J. Bioterrorism Biodefense.

[27] Bresk C.A., Hofer T., Wilmschen S., Krismer M., Beierfuß A., Effantin G., Weissenhorn W., Hogan M.J., Jordan A.P., Gelman R.S. (2019). Induction of tier 1 HIV neutralizing antibodies by envelope trimers incorporated into a replication competent vesicular stomatitis virus vector. Viruses.

[28] Ko C., Su J., Festag J., Bester R., Kosinska A.D., Protzer U. (2021). Intramolecular recombination enables the formation of hepatitis B virus (HBV) cccDNA in mice after HBV genome transfer using recombinant AAV vectors. Antivir. Res..

[29] Kosinska A.D., Moeed A., Kallin N., Festag J., Su J., Steiger K., Michel M.L., Protzer U., Knolle P.A. (2019). Synergy of therapeutic heterologous prime-boost hepatitis B vaccination with CpG-application to improve immune control of persistent HBV infection. Sci. Rep..

[30] Kosinska A.D., Festag J., Mück-Häusl M., Festag M.M., Asen T., Protzer U. (2021). Immunogenicity and antiviral response of therapeutic hepatitis B vaccination in a mouse model of HBeAg-negative, persistent HBV infection. Vaccines.

[31] Su J., Taji Z.H., Kosinska A.D., Oz E.A., Xie Z., Bielytskyi P., Shein M., Hagen P., Esmaeili S., Steiger K. (2024). Introducing adjuvant-loaded particulate hepatitis B core antigen as an alternative therapeutic hepatitis B vaccine component. JHEP Rep..

[32] Volz A., Sutter G. (2017). Modified vaccinia virus Ankara: History, value in basic research, and current perspectives for vaccine development. Adv. Virus Res..

[33] Acres B., Bonnefoy J.Y. (2008). Clinical development of MVA-based therapeutic cancer vaccines. Expert Rev. Vaccines.

[34] Truckenmiller M.E., Norbury C.C. (2004). Viral vectors for inducing CD8+ T cell responses. Expert Opin. Biol. Ther..

[35] Wilmschen S., Schneider S., Peters F., Bayer L., Issmail L., Bánki Z., Grunwald T., von Laer D., Kimpel J. (2019). RSV vaccine based on rhabdoviral vector protects after single immunization. Vaccines.

[36] Maini M.K., Pallett L.J. (2018). Defective T-cell immunity in hepatitis B virus infection: Why therapeutic vaccination needs a helping hand. Lancet Gastroenterol. Hepatol..

[37] Asabe S., Wieland S.F., Chattopadhyay P.K., Roederer M., Engle R.E., Purcell R.H., Chisari F.V. (2009). The size of the viral inoculum contributes to the outcome of hepatitis B virus infection. J. Virol..

[38] Aubert R.D., Kamphorst A.O., Sarkar S., Vezys V., Ha S.-J., Barber D.L., Ye L., Sharpe A.H., Freeman G.J., Ahmed R. (2011). Antigen-specific CD4 T-cell help rescues exhausted CD8 T cells during chronic viral infection. Proc. Natl. Acad. Sci. USA.

[39] Seder R.A., Hill A.V. (2000). Vaccines against intracellular infections requiring cellular immunity. Nature.

[40] Pulendran B., Ahmed R. (2011). Immunological mechanisms of vaccination. Nat. Immunol..

[41] Gressier E., Schulte-Schrepping J., Petrov L., Brumhard S., Stubbemann P., Hiller A., Obermayer B., Spitzer J., Kostevc T., Whitney P.G. (2023). CD4+ T cell calibration of antigen-presenting cells optimizes antiviral CD8+ T cell immunity. Nat. Immunol..

[42] Iwasaki A., Medzhitov R. (2010). Regulation of adaptive immunity by the innate immune system. Science.

[43] Barouch D.H. (2010). Novel adenovirus vector-based vaccines for HIV-1. Curr. Opin. HIV AIDS.

[44] Quigley M., Pereyra F., Nilsson B., Porichis F., Fonseca C., Eichbaum Q., Julg B., Jesneck J.L., Brosnahan K., Imam S. (2010). Transcriptional analysis of HIV-specific CD8+ T cells shows that PD-1 inhibits T cell function by upregulating BATF. Nat. Med..

[45] Lu S. (2009). Heterologous prime–boost vaccination. Curr. Opin. Immunol..

[46] Knolle P.A., Wohlleber D. (2016). Immunological functions of liver sinusoidal endothelial cells. Cell. Mol. Immunol..

[47] Maini M.K., Boni C., Ogg G.S., King A.S., Reignat S., Lee C.K., Larrubia J.R., Webster G.J., McMichael A.J., Ferrari C. (1999). Direct ex vivo analysis of hepatitis B virus-specific CD8(+) T cells associated with the control of infection. Gastroenterology.

[48] Thimme R., Wieland S., Steiger C., Ghrayeb J., Reimann K.A., Purcell R.H., Chisari F.V. (2003). CD8(+) T cells mediate viral clearance and disease pathogenesis during acute hepatitis B virus infection. J. Virol..

[49] Rehermann B. (2013). Pathogenesis of chronic viral hepatitis: Differential roles of T cells and NK cells. Nat. Med..

[50] Protzer U., Maini M.K., Knolle P.A. (2012). Living in the liver: Hepatic infections. Nat. Rev. Immunol..

[51] Lasaro M.O., Ertl H.C. (2009). New insights on adenovirus as vaccine vectors. Mol. Ther..

[52] Barouch D.H., Liu J., Li H., Maxfield L.F., Abbink P., Lynch D.M., Iampietro M.J., SanMiguel A., Seaman M.S., Ferrari G. (2012). Vaccine protection against acquisition of neutralization-resistant SIV challenges in rhesus monkeys. Nature.

[53] Wallace R., Bliss C.M., Parker A. (2024). L The Immune System-A Double-Edged Sword for Adenovirus-Based Therapies. Viruses.

[54] Kotturi M.F., Scott I., Wolfe T., Peters B., Sidney J., Cheroutre H., von Herrath M.G., Buchmeier M.J., Grey H., Sette A. (2008). Naive precursor frequencies and MHC binding rather than the degree of epitope diversity shape CD8+ T cell immunodominance. J. Immunol..

[55] Akondy R.S., Johnson P.L., Nakaya H.I., Edupuganti S., Mulligan M.J., Lawson B., Miller J.D., Pulendran B., Antia R., Ahmed R. (2015). Initial viral load determines the magnitude of the human CD8 T cell response to yellow fever vaccination. Proc. Natl. Acad. Sci. USA.

[56] Yewdell J.W., Bennink J.R. (1999). Immunodominance in major histocompatibility complex class I–restricted T lymphocyte responses. Annu. Rev. Immunol..

[57] Shin H., Wherry E.J. (2007). CD8 T cell dysfunction during chronic viral infection. Curr. Opin. Immunol..

[58] McLane L.M., Abdel-Hakeem M.S., Wherry E.J. (2019). CD8 T cell exhaustion during chronic viral infection and cancer. Annu. Rev. Immunol..

[59] Dion S., Bourgine M., Godon O., Levillayer F., Michel M.L. (2013). Adeno-associated virus-mediated gene transfer leads to persistent hepatitis B virus replication in mice expressing HLA-A2 and HLA-DR1 molecules. J. Virol..

