# Supplementary material for: Protein Priming Followed by a Replication-Competent VSV-GP Vector Boost Induces Sustained Immune Control in Therapeutic Hepatitis B Vaccination
Source: Vaccines (Basel). 2026 Mar 16;14(3):266. doi: 10.3390/vaccines14030266 (PMC13030091; doi:10.3390/vaccines14030266)
Supplement: Supplementary file 1 [file vaccines-14-00266-s001.zip › vaccines-4108293-supplementary.pdf]

vaccines

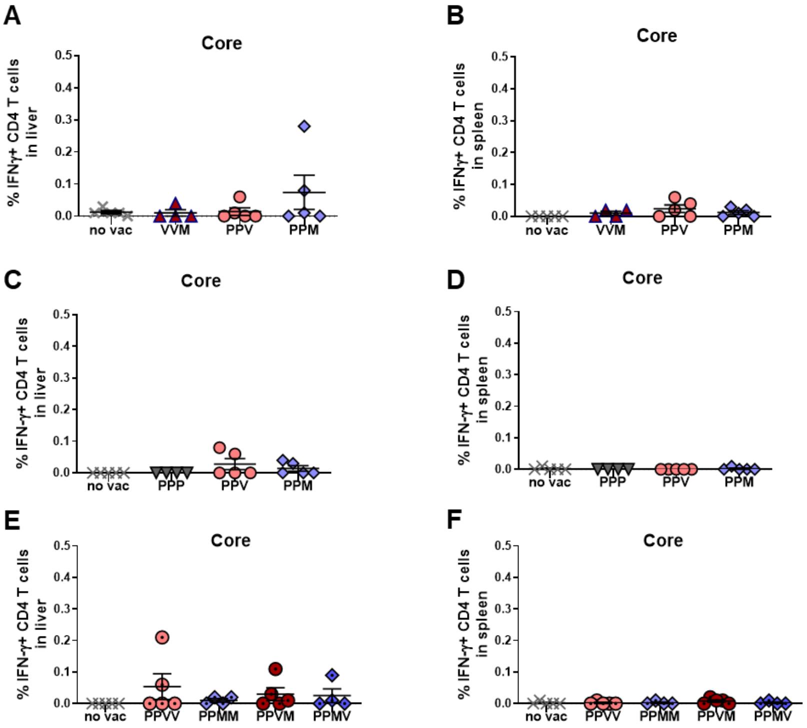

Supplementary Figure S1: HBV core-specific CD4 T-cell responses induced by the immunization with different VSV-GP-HBs/c-based heterologous prime-boost regimens in HBV-carrier mice. (A, B) Percentages of core-specific IFNγ+ CD4 T cells in liver (A) and spleen (B) were determined by ICS following stimulation with core-specific peptide pools in the experimental setting shown in Figure 3. (C, D) Percentages of core-specific IFNγ+ CD4 T cells in liver (C) and spleen (D) in the experimental setting shown in Figure 4. (E, F) Percentages of core-specific IFNγ+ CD4 T cells in liver (E) and spleen (F) in the experimental setting shown in Figure 5.

 https://doi.org/10.3390/vaccines14030266

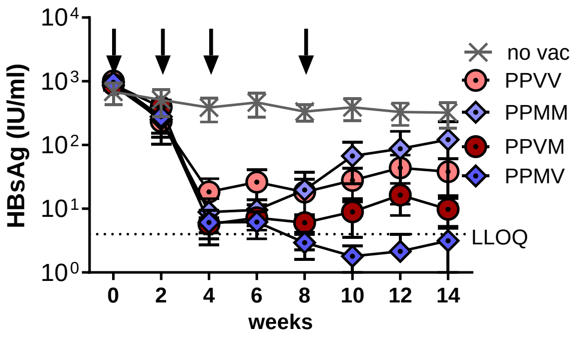

Supplementary Figure S2: Time kinetics of serum HBsAg levels throughout the experiment. C57BL/6 mice were intravenously transduced with 6×10^9 GE AAV-HBV1.2 virus six weeks prior to the initiation of the immunization to establish the persistent HBV infection. HBV-carrier mice were immunized intramuscularly with HBsAg and HBcAg adjuvanted with c-di-AMP at weeks 0 and 2. At weeks 4 and 8, mice received either homologous or heterologous booster immunizations with MVA-HBs/c or VSV-GP-HBs/c as indicated in the table. Six weeks after the 2nd boost immunization (week 14), mice were sacrificed to evaluate vaccine-induced immune responses. The serum levels of HBsAg during the experiment were monitored every 2 weeks. The arrows indicate the vaccination time points.

The original Western blot images of Figure 1B

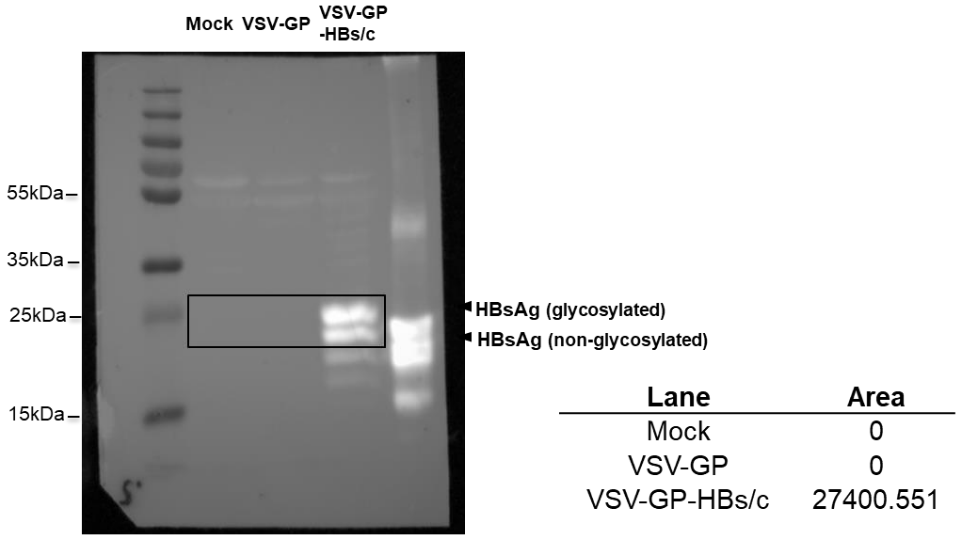

Lane	Area
Mock	0
VSV-GP	0
VSV-GP-HBs/c	27400.551

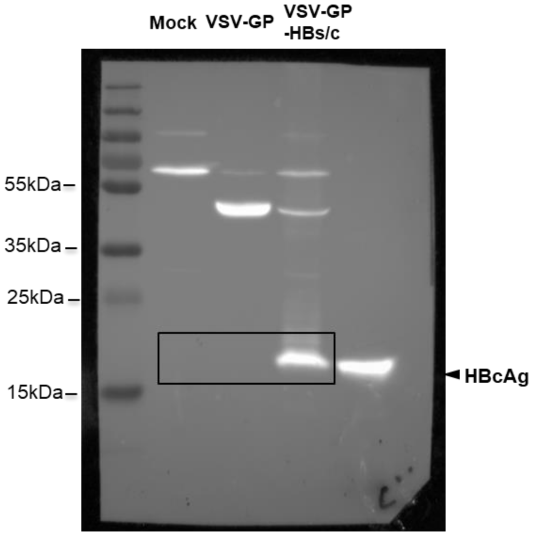

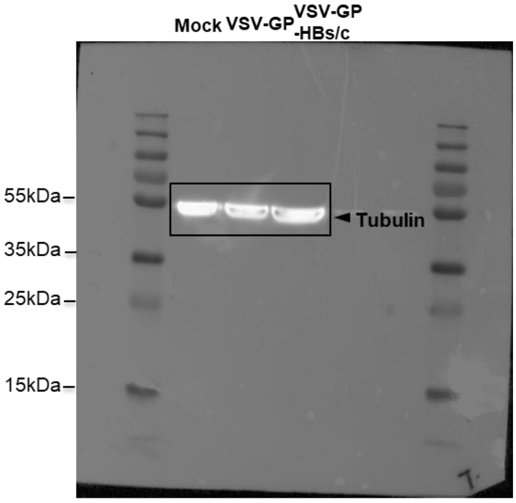

Lane	Area
Mock	0
VSV-GP	0
VSV-GP-HBs/c	19899.288

Lane	Area
Mock	32747.714
VSV-GP	30785.25
VSV-GP-HBs/c	27786.974

https://doi.org/10.3390/vaccines14030266
